# A novel upregulated hsa_circ_0032746 regulates the oncogenesis of esophageal squamous cell carcinoma by regulating miR-4270/MCM3 axis

**DOI:** 10.1186/s40246-023-00564-7

**Published:** 2024-01-10

**Authors:** Sachin Mulmi Shrestha, Xin Fang, Hui Ye, Lihua Ren, Qinghua Ji, Ruihua Shi

**Affiliations:** 1 Department of Gastroenterology, School of Medicine, Southeast University, Nanjing, Jiangsu Province China; 2 Department of Gastroenterology, Zhongda Hospital Affiliated to Southeast University, Nanjing, Jiangsu Province China

**Keywords:** circRNA, Esophageal squamous cell carcinoma, microRNA, Upregulated

## Abstract

**Introduction:**

Circular RNAs (CircRNA) have emerged as an interest of research in recent years due to its regulatory role in various kinds of cancers of human body. Esophageal squamous cell carcinoma (ESCC) is one of the major disease subtype in Asian countries, including China. CircRNAs are formed by back-splicing covalently joined 3′- and 5′- ends rather than canonical splicing and are found to have binding affinity with miRNAs that conjointly contribute to oncogenesis.

**Materials and methods:**

4 pairs of normal, cancer adjacent tissues and cancer tissues were analyzed by high-throughput RNA sequencing and 84 differentially upregulated circRNAs were detected in cancer tissues. hsa_circ_0032746 was silenced by siRNA and lentivirus and then further proliferation, migration and invasion were performed by CCK-8 and transwell assays. Bioinformatic analysis  predicted binding affinity of circRNA/miRNA/mRNA axis.

**Results:**

After qPCR validation, we selected a novel upregulated hsa_circ_0032746 to explore its biogenetic functions which showed high expression in cancer tissues but not in cancer adjacent tissues. The clinicopathological relation of hsa_circ_0032746 showed positive correlation with the tumor location (*P* = 0.026) and gender (*P* = 0.05). We also predicted that hsa_circ_0032746 could sponge with microRNA. Bioinformatic analysis predicted 11 microRNA response element (MRE) sequences of hsa_circ_0032746 and dual luciferase reporter assay confirmed binding affinity with miR4270 evidencing further study of circRNA/miRNA role. The knockdown of hsa_circ_0032746 by siRNA and lentivirus demonstrated that proliferation, invasion and migration of ESCC were inhibited in vitro and vivo experiments. Bioinformatic analysis further predicted MCM3 as a target of miR-4270 and was found upregulated in ESCC upon validation. miR4270 mimic decreased the level of hsa_circ_0032746 and MCM3 while further rescue experiments demonstrated that hsa_circ_0032746 was dependent on miR4270/MCM3 axis on the development process of ESCC.

**Conclusion:**

We revealed for the first time that circ_0032746/mir4270/MCM3 contributes in proliferation, migration and invasion of ESCC and could have potential prognostic and therapeutic significance.

## Introduction

Esophageal squamous cell carcinoma (ESCC), one of the subtypes of esophageal carcinoma, is a highly malignant tumor prevalent in Asian countries including China. There is upto ten-fold differences in the prevalence among the wide geographic distribution of countries. In China, ESCC is reported to occur combined with gastric cardia adenocarcinoma, however, the phenomenon of this combination is yet to be explored clearly [1]. Based on GLOBOCAN 2020 data, esophageal cancer ranks seventh in terms of incidence and sixth in mortality throughout the world. Whereas, in China it ranks fifth and fourth respectively [29]. Although, the gender-wise distribution is assumed to be more in male than woman but in China, the ratio even approached to 1:1 [2]. The pathogenesis evolves as a transitional phase of normal squamous epithelia to dysplasia to carcinoma. Various genetic alterations are accounted for its origination, progression and metastasis. The need of much detailed understanding of molecular pathogenesis is alarming to develop ESCC molecular targeted therapies in near future such that therapeutic contributions could be made to improve its poor prognosis.

Circular RNAs are non-coding endogenous RNAs possessing remarkable feature of non-canonical splicing without a free 3′ or 5′ end [10]. CircRNAs are categorized as exonic (E), intronic (I), and exon- intron (EI) as generated from the primary transcript sequence [16, 21, 34]. Recently, globally explored circRNAs have been found to be involved in the development of various types of diseases, such as atherosclerosis, nervous system disorders, Hirschsprung disease, osteosarcoma and various other carcinomas [3, 4, 7, 25]. They are also resistant to exonuclease making them more stable than linear RNA isoforms due to the lack of accessible ends. They are common in eukaryotes and can interact with microRNAs (miRNAs) or other molecules to regulate gene expression at the transcriptional or post-transcriptional level. The sponging of circRNA with miRNA protects target genes from miRNA-mediated mRNA degradation via the underlying mechanisms of competition for endogenous RNA (ceRNAs) [28, 36]. Hence, in this study, we explored a novel hsa_circ_0032746 derived by high-throughput sequencing and its role in the oncogenesis of ESCC. Furthermore, its regulatory role on sponging with miRNAs and modulating the target genes to understand the underlying mechanism in the progression of ESCC was also explored.

## Methods and materials

### Clinical specimens and cell lines

A total of 42 pairs of normal tissues, cancer adjacent tissues and esophageal squamous cancer tissues were obtained from Cardio-Thoracic surgery department and gastroenterology department of Southeast university affiliated Zhongda Hospital during 2018 to 2021 A.D (15 pairs of surgical specimens and 27 pairs of ESD specimens). Among them 28 were male and 14 were female with mean age of 65.45 ± 6.58 years old. All samples were frozen in liquid nitrogen until RNA total isolation. Normal esophageal epithelial tissue was defined as tissues that were ≥ 5 cm apart from the margin of the cancer tissues and tumor adjacent tissues were defined as tissues that were ≥ 2 cm and ≤ 5 cm apart from the margin of the cancer tissues. All patients with ESCC had not received any local or systemic treatment before operation and didn’t have any metastatic lesion. Approved guidelines were followed during collection and storing of specimens. This study was approved by the Research Ethics committee of the Southeast University (Nanjing, China; approval no. 2019ZDSYLL023‐Y01). All patients provided written informed consent.

Human normal esophageal epithelial cell line (HEEC) was purchased from Bena culture collection; Beina Chunglian Biotechnology Research Institute (Beijing, China) and esophageal squamous cancer cell lines (KYSE 30, KYSE 410, KYSE 150, KYSE 140, TE-1, TE-13 and ECA-109) were purchased from Cell cook company (Guangzhou, China) and National collection of authenticated cell culture (Shanghai, China). All cells were cultured in the cell culture medium (RPMI 1640, Basalmedia Technologies Co., Ltd, Shanghai, China) supplemented with 10% FBS (Cellcook Biotech Co., Ltd) and 1% antibiotics (streptomycin + penicillin) (Basalmedia Technologies Co., Ltd, Shanghai, China) at 5% CO_2_ at 37^0^C incubation. Cell lines were authenticated by STR DNA profiling analysis.

### RNA-sequencing

Next‐generation high-throughput RNA sequencing was performed from 4 patients’ tissue samples (paired normal, cancer adjacent and cancer) at Shanghai Kangchen Biotechnology co., Ltd. using Illumina HiSeq 4000 (Illumina, Inc.). The differentially expressed circRNAs and mRNAs were selected by log2fc (≥ 2.0), *P* value (*P* < 0.05) and false discovery rate (FDR < 0.05) as the thresholds. Image processing and base recognition were performed using Solexa pipeline version 1.8 software (Off‐Line Base caller software). Then, the reference genome was compared using Hisat2 software (version 2.1.0; http://daehwankimlab.github.io/hisat2/) [13] and a FPKM calculation at the gene and transcript levels was performed using R software ‘Ballgown’.

### Regulatory relationship prediction

CircRNA-miRNA regulatory relationship was predicted using miranda (ver 3.3), RNA hybrid and circular RNA interactome databases. To establish circRNA-miRNA network, we searched MREs on circRNAs then selected the miRNAs as per their seed match sequences. Target genes regulated by miRNAs were predicted using Targetscan, mirTarbase and miRanda databases (shown in Fig. 5A).

### Total RNA extraction and quantitative real-time polymerase chain reaction (qRT-PCR)

Total RNA was isolated from frozen tissues in liquid nitrogen and cell lines using RNA-easy isolation reagent kit (Vazyme, China) according to manufacturer’s instructions. RNA quantity and quality were evaluated by Nano Drop spectrophotometer (Nano Drop Thermo, Wilmington, DE). cDNA was synthesized by reverse transcription (RT) using Swescript RT I first strand cDNA synthesis kit (Servicebio, China). Divergent primers of hsa_circ_0032746 were synthesized by Generay company (Shanghai, China). β-actin and U6 were used as a reference gene. The primer sequence for β-actin were F: TGAGAGGGAAATCGTGCGTGAC and R: GCTCGTTGCCAATAGTGATGACC; hsa_circ_0032746 were F: CATCAAGAGGAAGCGGAAAC and R: AGGGGATTGAACTCATGTGC; miR-4270 were F: CGGGCTCAGGGAGTCAGG and R: CAGCCACAAAAGAGCACAAT, Stem loop Primer was GTCGTATCCAGTGCAGGGTCCGAGGTATTCGCACTGGATACGACGCCCTC; U6 were F: CTCGCTTCGGCAGCACA and R: AACGCTTCACGAATTTGCGT; MCM3 were F: CTTTCCCTCCAGCTCTGTCTA and R: TCACCAGGCTTCGCTTTATC. The expression level of the circRNAs was evaluated by qPCR using ChemQ Universal SYBR Green mix (Vazyme, China) following manufacturer’s instructions. Both target and reference genes were amplified in triplicate wells. And the relative level of each circRNA was calculated using 2^−△△Ct^ method.

### Transfection with siRNA and shRNA

Two Cell lines, kyse30 and TE-1 were transfected with three different sequences of si-circ0032746, synthesized by Genepharma (Shanghai, China), for 48 h using Lipofectamine 3000 (Invitrogen) and the knockdown efficiency was validated by qRT-PCR. The three different sequences used for the si-circ_0032746 target sequence are shown below:

siRNA1: AGUGCAGGAGACCAGCAUATT (sense) and UAUGCUGGUCUCCUGCACUTT (antisense);

siRNA2: GUGCAGGAGACCAGCAUAATT (sense) and UUAUGCUGGUCUCCUGCACTT (antisense);

siRNA3: GCAGGAGACCAGCAUAAACTT (sense) and GUUUAUGCUGGUCUCCUGCTT (antisense).

Then, the lentivirus-mediated GV248 vector expressing si-circ0032746 used for knockdown was constructed and packaged to express short hairpin RNA (shRNA) to knockdown hsa_circ_0032746 by Genepharma (Shanghai, China). Cells were transfected by lentiviral particles when the cells growth reached to 40–50% confluency. The medium was replaced after 24 h of infection and the cells were screened by adding puromycin (5 μg/ml) for stable transfection. The sequence of sh-circ0032746 is: GCAGGAGACCAGCATAAAC.

### RNase R treatment assay

Total of 2 µg RNA was diluted in 20 μl of water and 4 units of RNase R (Epicenter) were added together to incubate for 30 min at 37 °C. Then, hsa_circ_0032746 and c14orf118 mRNA expression was detected through qRT-PCR after RNase R treatment.

### Subcellular localization

The nuclear or cytoplasmic subcellular localization of hsa_circ_0032746 was detected using the PARIS Kit (Invitrogen, Carlsbad, CA, USA) in accordance with the manufacturer’s instructions.

### Cell proliferation assay

The proliferation of two selected cell lines was detected by the Cell Counting Kit-8 (CCK-8) assay (cell cook, China). 100 ul of cell suspension containing 5 × 10^3^ cells were added into each well of the 96-well plate and the absorbance was measured at 450 nm at 0 h, 24 h, 48 h, 72 h and 96 h interval of time after transfection, respectively. For colony formation assay, 2300 cells/well were plated into the 6-well plate. After 14 days, the colonies were fixed, stained and counted.

### Flow cytometry analysis for cell cycle and apoptosis

Cells were harvested after transfection. Cell cycle was detected by labelling cells with PI (Beyotime, China). Apoptosis of cells were detected using Annexin V-fluorescein isothiocyanate/ propidium iodide apoptosis detection kit (BD science). The protocol was followed as per the manufacturers’ instructions for both the analyses. Cells labeled with FITC-labeled Annexin V and PI were measured by flow cytometry.

### Transwell assays

Two cell lines were plated into the upper chamber (8 μm pore size, Falcon, USA) of the plate with or without Matrigel (BD Biosciences) at a density of 5.5 × 10^4^/well. Then, the upper chamber was filled with the serum-free culture solution, while the lower chamber was filled with RPMI-1640 containing 10% FBS to chemoattract the cells from the upper chamber. 48 h later, the migrated cells were fixed with ethanol and stained with 1% crystal violet. To analyze the invasion capability of the cells, upper chamber was paved with matrigel (Invitrogen). The results were imaged and counted in inverted microscope.

### Western blot

Cells were lysed in lysis buffer containing phosphatase inhibitors and the concentrations of protein were obtained using bicinchoninic acid (BCA) method (Fdbio science, China). A total of 18 μg of protein was separated by using 10% SDS–PAGE gel and transferred onto polyvinylidene difluoride (PVDF) membrane (Merck Millipore, Ireland). After blocking for 1–2 h, the membrane was incubated with primary antibody anti-MCM3 (ab-DF6205, Affinity Biosciences) and anti-B-actin (20536-1-AP, Proteintech Group Inc.) diluted in 5% BSA or skimmed milk in TBST with shaking overnight at 4 °C followed by HRP conjugated secondary antibody at room temperature for 2 h. B-actin was used as the loading control. The protein expression was detected by using enhanced chemiluminescence (Biosharp, China).

### Dual-luciferase reporter assay

Wild-type hsa_circ_0032746-wt or Mutant hsa_circ_0032746-mut was inserted into a dual-luciferase reporter vector (GP-mirGLO). 5 × 10^4^ Kyse450 cells/well were plated in 24-well plate for 24 h before transfection. The cells were co-transfected with the dual-luciferase reporter plasmid and miR-4270 NC or miR- 4270 mimics and miR- 4270 NC inhibitor or miR-4270 inhibitor by the Lipofectamine 3000 (Invitrogen). After 48 h, we measured the relative luciferase activity by Dual-luciferase Reporter assay system (Promega, Madison, WI, USA).

### Xenograft model

Four to five weeks old BALB/c nude male mice were purchased from Cavens experiment animal model Company (Changzhou, China) and housed under pathogen-free conditions. KYSE30 cells (2–3 × 10^6^) were injected into the dorsal flank of each mouse subcutaneously and tumor volumes were measured every 7 days with electronic calipers. Forty five days after inoculation, the animals were euthanized and tumor samples were collected. The experiment was performed in accordance with the institutional ethics guidelines for animal experiments, approved by the animal management committee of Southeast University (Nanjing, China; approval no. 20210709007).

### Statistical analysis

The variable data were analyzed by chi-square test using SPSS 22.0 software (IBM, USA). Unpaired 2-tailed Student’s t-tests were conducted for comparisons between 2 groups, and one- way ANOVA with Tukey’s post hoc test was performed to compare more than 2 groups and data analysis was conducted using GraphPad Prism 8.2. All experiments were repeated at least three times, and differences with *P* < 0.05 were considered significant.

## Results

### hsa_circ_0032746 expression level was upregulated in human ESCC tissues and cell lines

**Fig. 1:**
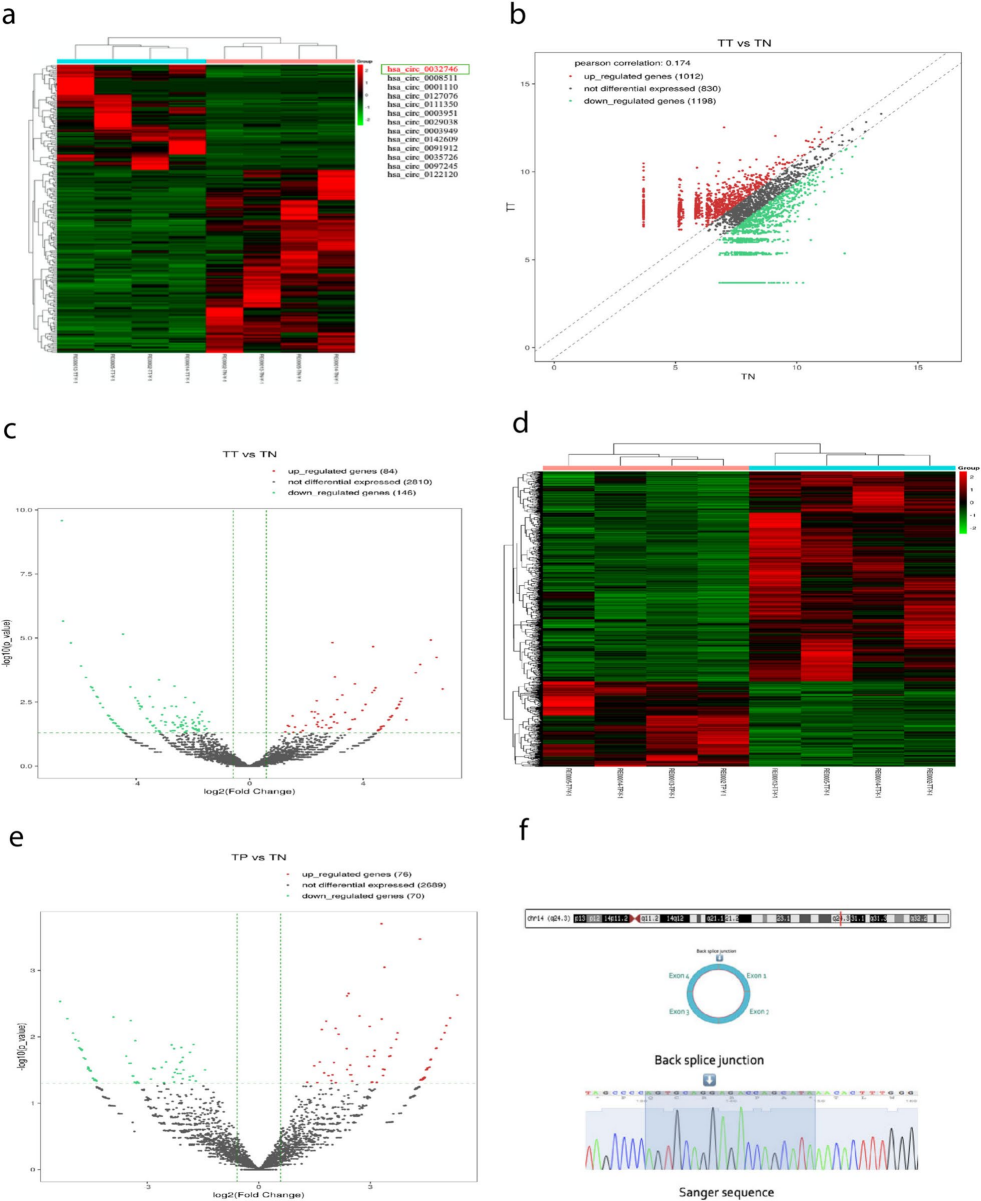
Heat maps illustrating expression profile of circRNA and its biological properties. **a** Heat map illustrates normalized expression values of significantly expressed circRNAs in cancer tissues with log2FC ≥ 0.585, *P* value cut-off ≤ 0.05, q-value cut-off ≤ 1.00. Each column represents one tissue sample. Red represents upregulated and green represents downregulated circRNAs. **b** Scatter plot illustrates differentially expressed circRNAs in cancer tissues. **c** Volcano graph illustrates differentially expressed circRNAs in cancer tissues with log2 fold change. **d** Heat map illustrates normalized expression values of significantly expressed circRNAs in cancer adjacent tissues with log2FC ≥ 0.585, p-value cut-off ≤ 0.05, q-value cut-off ≤ 1.00. Each column represents one tissue sample. Red represents upregulated and green represents downregulated circRNAs. **e** Volcano graph illustrates differentially expressed circRNAs in cancer adjacent tissues with log2 fold change. **f** hsa_circ_0032746 characterization figure. hsa_circ_0032746 is back-spliced by exons 1 and 4. Back splice junction obtained by Sanger sequencing. The chromosomal location of hsa_circ_0032746

**Fig. 2:**
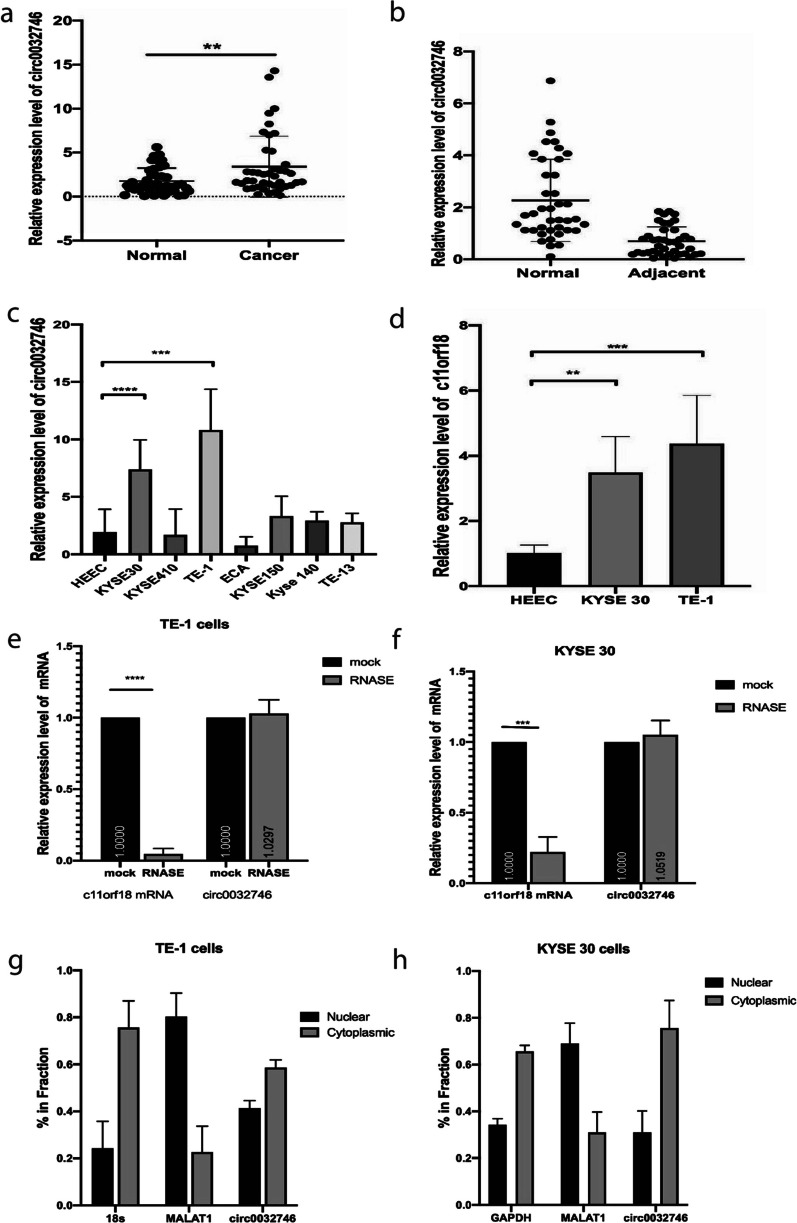
Expression level of hsa_circ_0032746 in cancer tissues and cell lines. **a** Higher expression of hsa_circ_0032746 in 42 pairs of esophageal cancer tissues compared to their matched normal tissues. **b** Expression level of hsa_circ_0032746 in 42 pairs of esophageal cancer adjacent tissues compared to their matched normal tissues. **c** Higher expression level of hsa_circ_0032746 in KYSE30 and TE-1 cell lines compared to others after normalizing to HEEC. **d** Higher expression level of c14orf118 in KYSE30 and TE-1 cell lines. **e** and **f** resistance of circular RNA to RNase R digestion in ESCC cell line. **g** and **h** Percentage of hsa_circ_0032746 expression level in nucleus and cytoplasm

4 pairs of normal and cancer tissues were analyzed by high-throughput RNA sequencing and total of 3040 circRNAs were detected (shown in Fig. 1a). Among which, 1012 were upregulated, 1198 were downregulated and 830 were non-differentially expressed circRNAs (shown in Fig. 1b). Furthermore, statistically significant differential expression revealed 84 upregulated circRNAs (log2FC ≥ 0.585, *p* value cut-off ≤ 0.05, q-value cut-off ≤ 1.00) (shown in Fig. 1c) (data accessible at NCBI GEO database, accession GSE89143). For further validation, we selected top 13 circRNAs by qPCR (log2FC ≥ 2.0). Then, after initial validation in 9 paired tissue samples and cancer cell lines, we selected a novel hsa_circ_0032746 as our circRNA of interest, based on its significant expression. We further validated the expression of hsa_circ_0032746 in total of 42 pairs of tissues, which showed significant high expression (P value = 0.0063) in cancer tissues than in normal tissues by qRT-PCR (shown in Fig. 2a). Also, KYSE 30 and TE-1 cancer cell lines demonstrated the highest expression and were selected for further in vitro studies (shown in Fig. 2c). Also, the correlations with the clinicopathological factors were analyzed, which demonstrated that expression of hsa_circ_0032746 was markedly correlated with location of the lesion (*P* = 0.026) and gender (*P* = 0.05). However, we didn’t find any significant correlation with other clinicopathological factors such as age, lesion size, treatment modality, differentiation, tumor invasion and nodal involvement (*P* > 0.05) (shown in Table 1).

**Table 1:** The correlation between hsa_ circ_0032746 expression and clinicopathological characteristics of 42 ESCC patients

Clinicopathological characteristics	Cases (n)	circ_0032746 expression		*P* value
		High (n = 21)	Low (n = 21)	
Age				
< 65	19	10	9	0.757
> 65	23	11	12	
Gender				
Male	28	17	11	0.05*
Female	14	4	10	
Lesion size				
≤ 5 cm	34	19	15	0.238
> 5 cm	8	2	6	
Treatment				
ESD	27	16	11	0.107
Esophagectomy	15	5	10	
Differentiation				
Well	14	7	7	
Moderate	21	10	11	0.909
Poor	7	4	3	
Location				
Upper	4	0	4	
Middle	17	12	5	0.026*
Lower	21	9	12	
Tumor invasion				
Tis,T1,T2	22	8	14	
T3,T4	20	13	7	0.126
Nodal involvement				
Positive	18	11	7	0.212
Negative	24	10	14	

### hsa_circ_0032746 expression level in cancer adjacent tissues

High-throughput RNA sequencing detected a total of 2835 circRNAs (shown in Fig. 1d). Among which,76 upregulated and 70 downregulated differentially expressed circRNAs (log2FC ≥ 0.585, p-value cut-off ≤ 0.05, q-value cut-off ≤ 1.00) were detected (shown in Fig. 1e). We also validated hsa_circ_0032746 in 42 pairs of normal and cancer adjacent tissues but found that hsa_circ_0032746 was not upregulated in cancer adjacent tissues compared to normal tissues (shown in Fig. 2b).

### The biological characteristics of hsa_circ_0032746

hsa_circ_0032746 is an annotated circRNA derived from c14orf118 mRNA located at q24.3 region of chromosome 14 containing exon 1,2,3,4. We performed Sanger sequencing to determine the back-splicing nature of circ_0032746 (Shown in Fig. 1f). The expression level of c14orf118 mRNA was found to be high in both the cell lines, KYSE30 and TE-1 (shown in Fig. 2d). When treated by RNASE R, the expression level of hsa_circ_0032746 didn’t show significant decrease whereas drastic decrease was noted in linear c14orf118 mRNA level, such that the stability of hsa_circ_0032746 was demonstrated (shown in Fig. 2e and f). Then, nuclear and cytoplasmic subcellular fraction experiment was performed which demonstrated that hsa_circ_0032746 was mainly located in the cytoplasm (shown in Fig. 2 g and h).

### Knockdown of hsa_circ_0032746 after siRNA and lentivirus transfection

**Fig. 3:**
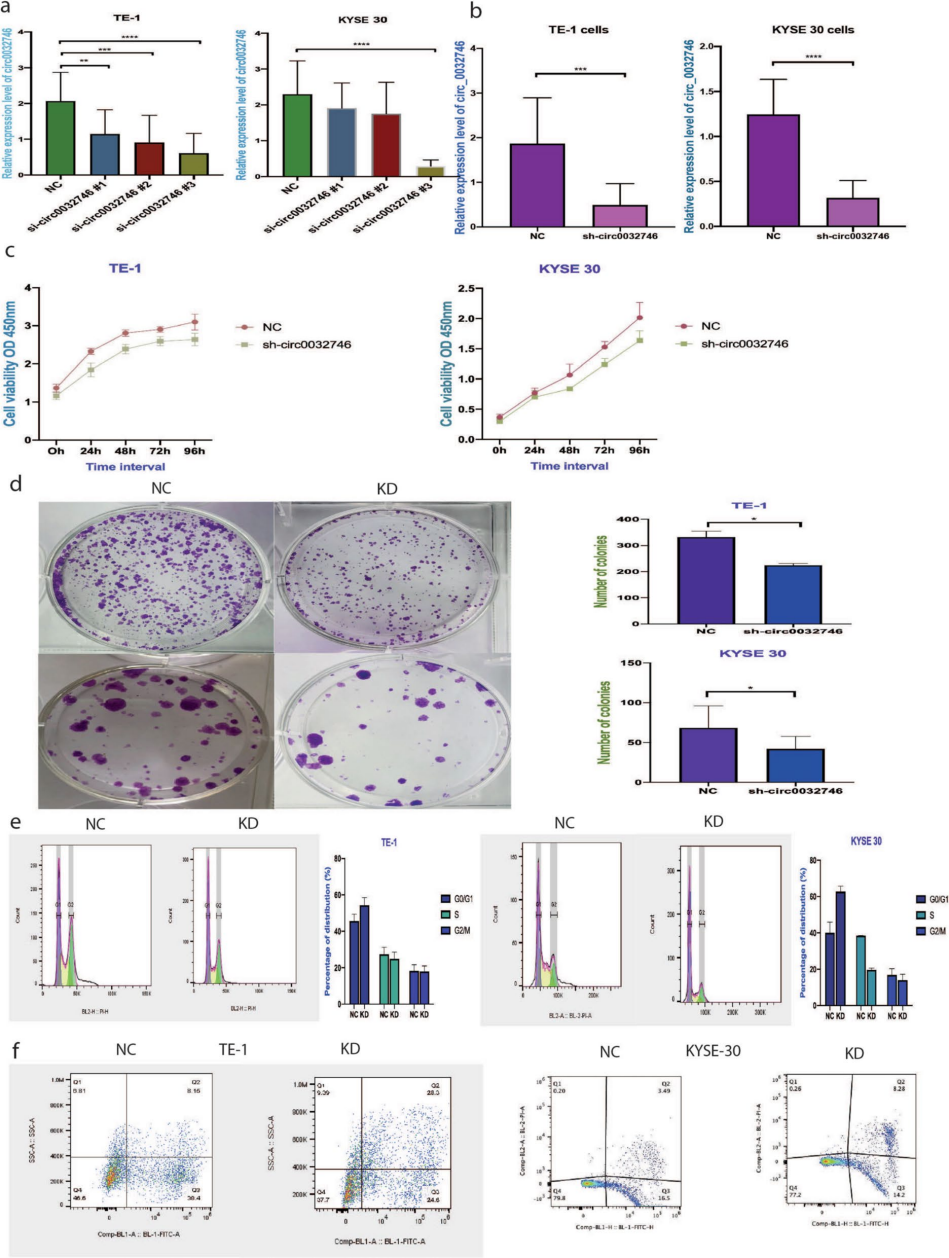
hsa_circ_0032746 promotes the oncogenesis of ESCC. **a** and **b** TE-1 and KYSE 30 cell lines transfected with siRNAs and shRNA and expression level validated by qRT-PCR. Lentivirus transfection induces inhibition of proliferation of cancer cells. **c** CCK8 assay. **d** colony formation assay. **e** Lentivirus transfection arrested cells at S phase. **f** More apoptotic cells seen in KD group than in NC group

As, RNA-seq results and our qPCR validation showed that hsa_circ_0032746 was highly expressed in cancer tissues and cancer cell lines, we transfected the cells via siRNA after which the expression was observed to be lower than control group. Hence, the silencing of hsa_circ_0032746 was achieved by RNA interference revealing its potential tumor-suppressive function. Among, the three sequences of siRNA, the sequence with the lowest expression was selected for further evaluation of knockdown efficiency (shown in Fig. 3a). Furthermore, shRNA induced knockdown of hsa_circ_0032746 also demonstrated remarkable lower expression in both the cancer tissues and cell lines (shown in Fig. 3b). The above results showed that both siRNA and shRNA could remarkably inhibit the expression of hsa_circ_0032746.

### Prediction and annotation of hsa_circ_0032746 targeted miRNA and mRNA network

**Fig. 4:**
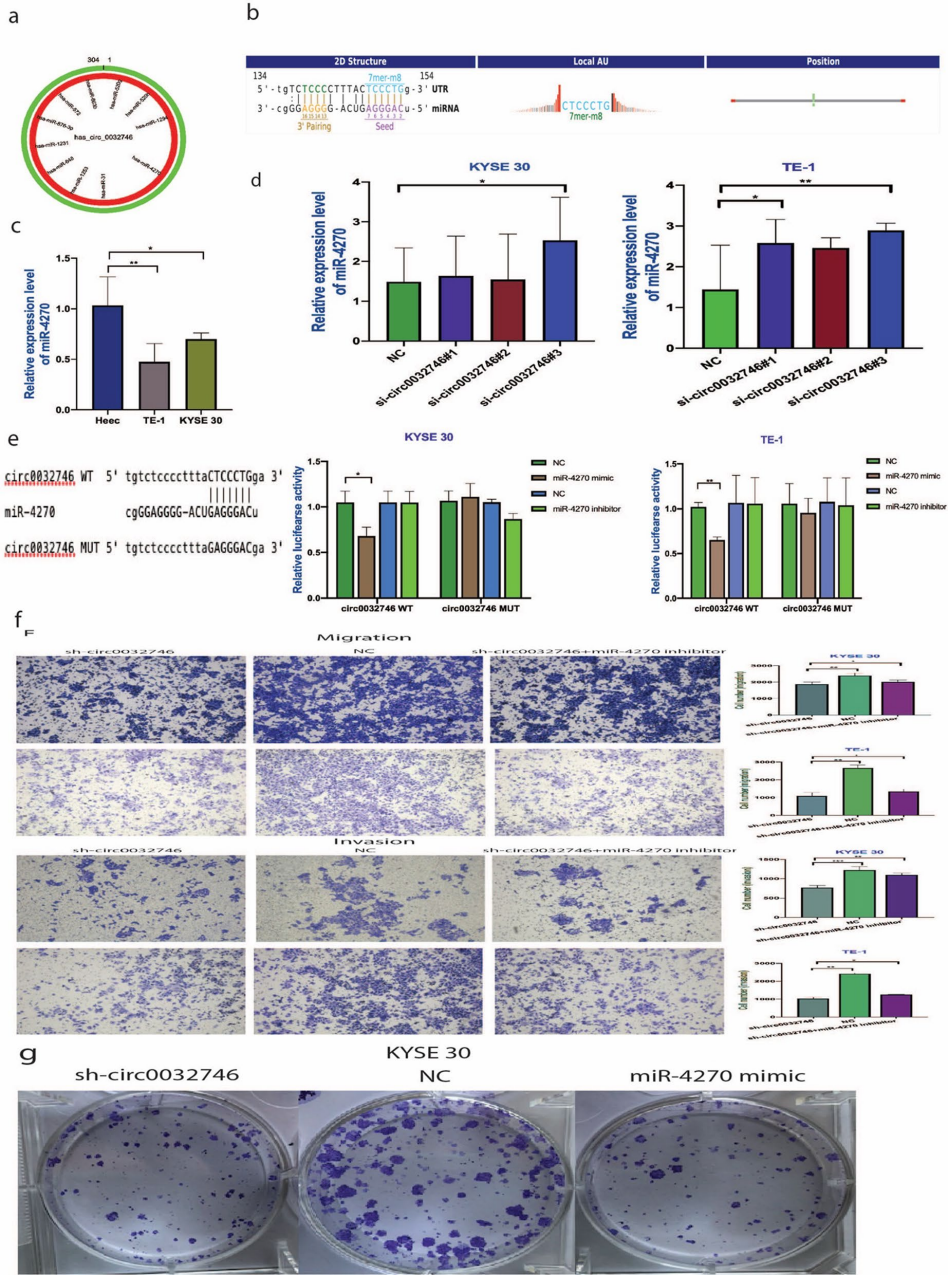
hsa_circ_0032746 serves as miR-4270 sponge. **a** The MREs of hsa_circ_0032746. **b** The binding sites for miR-4270 and hsa_circ_0032746. **c** The expression level of miR4270 in TE-1 and KYSE 30 cell lines. **d** The expression level of miR4270 in TE-1 and KYSE 30 cell lines after siRNA transfection. **e** Prediction of combined sequence of hsa_circ_0032746 WT and miR‐4270 and construction of hsa_circ_0032746 MUT sequence. The luciferase intensity decreased after cotransfection with hsa_circ_0032746 wild-type and hsa- miR-4270 mimics in both cell lines. **f** Transwell assay illustrated that lentivirus transfection induced knockdown of hsa_circ_0032746 in both cell lines whereas cotransfection with miR-4270 inhibitor reversed the effect. **g** Colony formation assay showed that sh-circ_0032746 and miR-4270 mimic inhibited the cell proliferation

**Fig. 5:**
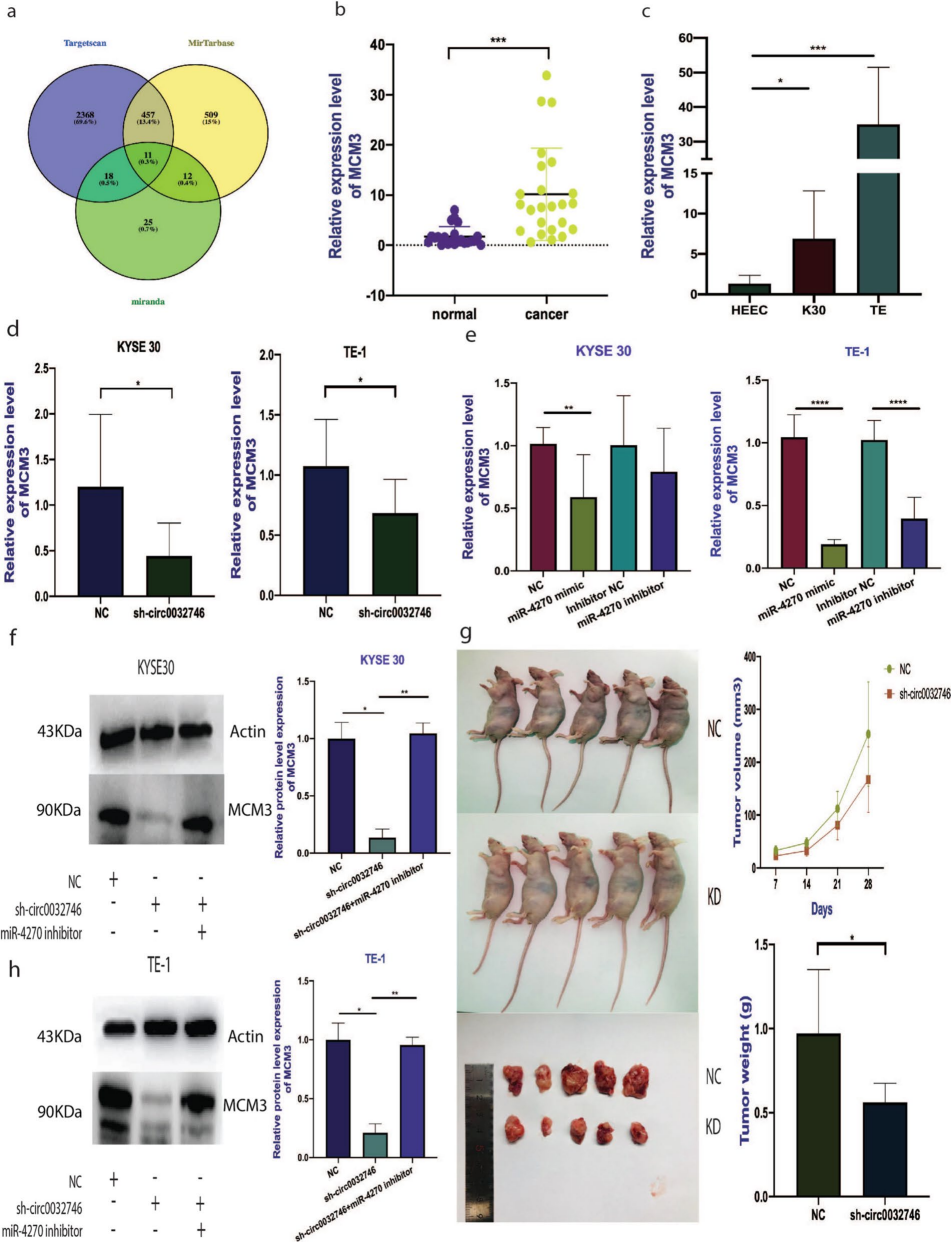
The expression level of MCM3 target gene of miR-4270. **a** Predicted targeted genes of miR-4270 by bioinformatic analysis. **b** and **c **Expression level of MCM3 in cancer tissues and KYSE30 and TE-1 cell lines. **d** Decreased expression level of MCM3 after knockdown by lentivirus. **e** The expression level of MCM3 after transfection with miR4270 mimic and inhibitor. **f** and **h** The protein level of MCM3 measured by western blot. **g** Intravenous injection of NC and sh-circ0032746 transfected KYSE30 cells into nude mice

To further determine the function of circRNAs, hsa_circ_0032746 sponging MREs were predicted by conserved seed-matching. Altogether, 11 microRNAs were found to sponge with hsa_circ_0032746 by analyzing the three databases (shown in Fig. 4a). Among which, we selected 4 microRNAs for validation to determine the regulation with hsa_circ_0032746. Then, based on the expressions on KYSE30 and TE-1 cell lines, we selected miR-4270 to further determine its binding ability (shown in Fig. 4b). Corresponding to the level of hsa_circ_0032746, our results indicated that miR-4270 expression was down-regulated in Kyse30 and TE-1 cells (shown in Fig. 4c) and up-regulated in cells treated with si-circ_0032746 (shown in Fig. 4d). Furthermore, to determine the interaction between hsa_circ_0032746 and miR‐4270, we performed dual-luciferase reporter assay by constructing wild type and mutant plasmids and transfected to two cell lines (shown in Fig. 4e). Our results showed that miR-4270 mimics considerably reduced the luciferase intensity of wild type hsa_circ_0032746 compared with mutant hsa_circ_0032746 (shown in Fig. 4e). Also, miR-4270 mimic transfection inhibited the KYSE 30 cell proliferation compared to NC group (shown in Fig. 4g). Then, miR-4270 binding target genes were predicted (shown in Fig. 5a) and the efficacy of sh-circ_0032746 and miR-4270 mimic and inhibitor was evaluated in miR-4270 binding target genes. Initially, altogether 9 target mRNAs (TNP1, FADS2, NRAS, PRIMA, TRIM15, AGO1, WNT3A, METTL1, MCM3) were selected to evaluate the binding efficiency as miR-4270 may negatively regulate target mRNA expression. On further validation, MCM3 was finally selected and its expression in esophageal cancer was analyzed in TCGA database which showed high expression in esophageal cancer tissues compared to normal tissues. In our further study, MCM3 was found to be highly expressed in cancer tissues and KYSE30 and TE-1 cell lines (shown in Fig. 5b and c) and its expression was decreased in cell lines transfected by sh-circ0032746 (shown in Fig. 5d). As, miR-4270 may negatively regulate MCM3 expression, miR-4270 mimic was transfected in cell lines which showed decreased expression of MCM3 whereas reversed on transfection with miR-4270 inhibitor (shown in Fig. 5e). Hence, the results suggested that hsa_circ_0032746 could upregulate the expression of MCM3 by sequestering miR‐4270.

### Knockdown of hsa_circ_0032746 inhibits cell proliferation

Cell proliferations have been analyzed using CCK-8 assay. KYSE 30 and TE-1 cells were incubated in 96-well plates after transfection by NC and sh-circ0032746. After 48 h, 72 h and 96 h, proliferation of cells in sh-circ0032746 groups were significantly lesser than those in NC group (shown in Fig. 3c). Colony formation assay also demonstrated that knockdown inhibited the proliferation of both cell lines (shown in Fig. 3d). Hence, results indicated that lentiviral vectors with shRNA sequence effectively inhibit the proliferation abilities of both the cell lines.

### Knockdown of hsa_circ_0032746 inhibits cell migration and invasion

Transwell assay showed that after transfection via sh-circ0032746, the migration and invasion ability of the cells were remarkably reduced as compared to the NC group. Similarly, co-transfection of miR-4270 inhibitor with sh-circ0032746 reversed the function of lentivirus knockdown (shown in Fig. 4f).

### Knockdown of hsa_circ_0032746 leads to cell cycle arrest and facilitates cell apoptosis

To analyze the efficiency of sh-circ0032746 on inhibiting cell proliferation, flow cytometry was performed on propidium iodide stained cells. As shown in Fig. 3e, knockdown of both TE-1 cells and KYSE 30 cells resulted in higher population of cells in G0/G1 phase compared to negative control. But, lower population of cells were noted in S phase of knockdown cells. Hence, these results revealed that transfection with sh-circ0032746 led to cell cycle arrest. Cell apoptosis was increased in two cell lines after transfected with sh-circ0032746. As shown in Fig. 3f, early and late apoptotic cells in knockdown group were significantly increased compared to the NC group.

### Knockdown of hsa_circ_0032746 inhibits xenotransplanted tumor growth of nude mice

2 * 10^6^ cells were injected in flank of the mice (NC and sh-circ0032746 groups). Tumors were allowed to grow for 5 weeks and then sacrificed. Tumor size and weight were recorded in every 7 days till the mice were sacrificed. All the mice were dissected but no metastatic tumor was found in other organs such as lung, liver or stomach. There were significant difference between size and weight among two groups as both the weight and volume in knockdown group were lower than the control group (as shown in Fig. 5g).

### Protein expression after lentivirus transfection

As expected, this experiment demonstrated decreased protein levels after circ_0032746 knockdown via lentivirus transfection in both cell lines. Rescue experiment demonstrated that miR-4270 inhibitor co-transfection significantly reversed the inhibitory effect of lentivirus circ_0032746 silencing such that protein expression was increased on co-transfection of sh-circ0032746 + miR-4270 inhibitor (as shown in Fig. 5f and h).

## Discussion

CircRNAs were observed in RNA viruses as early as the 1970s [32, 33]. Then, as time passed by, state-of the-art techniques such as high-throughput sequencing and bioinformatic analysis further contributed to identify more numbers of circRNAs. As, circRNAs may arise from exons or introns so both exonic and intronic circRNAs have potential functions in the regulation of gene expression [35]. On further study, researchers have found the role of circRNAs as promotors or inhibitors in various types of cancer. Although, the roles of circRNAs are yet to be further explored in the cancers, highly conserved sequences and a high degree of stability make them structurally and functionally unique than its counterpart in molecular study [9, 12, 26]. Since, circRNAs are differentially expressed in human cells and tissues, they can be multiple folds higher compared to their linear isomers. Cao et.al found circRNA_100876 was upregulated in cancer tissues of 74 patients with ESCC and in Eca-109 and TE-1 cancer cell lines [5]. Similarly, another research by Li et al. studied circITCH in 358 and 326 paired ESCC samples and found high expression in the tissues and Eca-109 and TE-1 cancer cell lines [17]. In this research, we identified 84 differentially upregulated circRNAs in cancer tissues and 76 differentially upregulated circRNAs in cancer adjacent tissues compared to normal. Then, after validation, we selected a novel hsa_circ_0032746 for further exploration of its biogenesis in cancer tissues, cell lines and cancer adjacent tissues and we believe our research is the first to report hsa_circ_0032746 to the best of our knowledge. The clinicopathological relation of hsa_circ_0032746 showed that tumor location (*P* = 0.026) and gender (*P* = 0.05) were positively correlated. Also, functional experiments found out that hsa_circ_0032746 can augment the proliferation, migration and invasion of esophageal squamous cancer cells and decrease the apoptotic rate. On the contrary, knockdown of hsa_circ_0032746 by lentivirus transfection inhibited the proliferation, migration and invasion, has induced cell cycle arrest and increased the apoptotic rate of the cancer cells. Additionally, in vivo study, the knockdown of hsa_circ_0032746 also revealed the growth inhibition of cancer in nude mice compared to control group. Furthermore, we also validated cancer adjacent tissues to observe field cancerization. As, many researchers have discussed the concept of field cancerization proposing that normal tissue adjacent to the cancer may have specific molecular characteristics which can eventually harbor the local recurrence or second primary tumors [28]. The reasons behind this concept were that mucosa adjacent to tumors was also observed molecularly abnormal and multifocal areas of precancerous changes develop due to widespread and protracted exposure to carcinogens. This concept has been applied to various types of cancers, especially squamous cell carcinomas of the head and neck, squamous cell carcinomas of the esophagus, adenocarcinomas from Barrett’s esophagus, gastric cancers, breast cancers and skin cancers [6, 8, 20, 24]. Vidal et al. performed RNA-Seq on 8 normal, matched cancer-adjacent gastric tissue and gastric tissue samples and speculated that the cancer-adjacent tissue presented significant number of circular RNAs and could not be considered as a normal tissue, reinforcing the notion of field cancerization (41). Similarly, our high-throughput sequencing also revealed 76 upregulated circRNAs compared to normal tissue. But, on qPCR validation of hsa_circ_0032746, it was not upregulated in cancer adjacent tissues in our study.

Then, we predicted circRNA/miRNA interactions based on conserved seed sequence matches. MicroRNAs are small (18-22nt) ncRNA molecules involved in posttranscriptional regulation of gene expression. It can regulate various cellular events such as cell proliferation, differentiation, migration and apoptosis [14, 19, 21–23, 37]. Additionally, miRNAs can also form the RNA-induced silencing complex (RISC) with Argonaute protein to silence or degrade the target mRNA. Numerous circRNA exploration researches indicated that circRNAs also play a crucial role in gene expression regulation by sponging with microRNA and inhibiting its activity. In our study, bioinformatic analysis of ceRNAs predicted that hsa-circ-0034726 has MREs of 11 oncogenic microRNAs. Among which, miR-4270 binding affinity was confirmed by dual-luciferase reporter assay and selected. Furthermore, rescue experiments found out hsa_circ_0032746 was dependent on miR-4270. Zhong et al. revealed that overexpression of circTCF25 downregulates miR-103a-3p and miR-107 and could regulate circTCF25-miR- 103a-3p/miR-107-CDK6 pathway in urinary bladder cancer, hence, promote proliferation and migration both in vivo and vitro [38]. Huang H et.al demostrated that ciRS-7 via miR-7/KLF4 and NF-κB signals could trigger the migration and invasion of ESCC [11]. Lin C et.al showed that the upregulated circRNA TCFL5 promoted the progression of ESCC by modulating M2 macrophage polarization via the miR-543-FMNL2 axis [15]. Zhou et al. demonstrated that the circPDE3B/miR-4766-5p/LAMA1 axis functions as an oncogenic factor in promoting ESCC by inducing EMT indicating its potential prognostic and therapeutic significance [36]. Various researchers have found different expressions and regulatory relationships of miR-4270 in various types of cancers. Wang et al*.*suggested that miR-4270 was upregulated in liver cancer and SATB2 expression was negatively correlated with miR-4270-5p levels in HCC [31]. Zhou D et.al also suggested that has_circ_0034326/miR-4270/*NRAS* and hsa-circ-0011950/miR-4270/ *NRAS* axes have regulatory relationships in HCC [39]. Shen D et.al studied hsa_circ_0005556/miR-4270/*MMP19* axis and revealed that this axis was involved in proliferation, migration, and invasion of gastric cancer [27]. Hence, we also further studied the ability of hsa_circ_0032746 to competitively bind with miR-4270 which could exert inhibitory effect on its target genes as well as circRNA. The relative expression of miR-4270 was low in TE-1 and KYSE30 cells lines. As, KYSE 30 and TE-1 cells were transfected with si-circ0032746, the expression level became higher.

MCM3, a target gene of miR-4270 was found to be upregulated in ESCC by our high-throughput sequencing data (GSE189830) suggesting it might act as an oncogene in the development process of ESCC. Additionally, bioinformatic analysis and our validation experiments revealed that MCM3 is a target gene of miR-4270. The role of MCM3 also has been studied in various cancers. Løkkegaard et al. [18] revealed that estrogen receptor-positive breast cancer cells could survive letrozole and tamoxifen treatments through upregulation of MCM3. Zhou H. et.al found that MCM3 was overexpressed in colorectal cancer cell lines and down-regulation inhibited proliferation, migration, invasion and promoted apoptosis [40]. Valverde et al. [30] indicated that MCM3 may emerge as a novel marker of cellular proliferation in Oral Squamous Cell Carcinoma. In our study based on the hypothesis that circRNA can act as a ceRNA to regulate miRNA target gene expression, we revealed that the expression of MCM3 was upregulated in cancer tissues and cancer cell lines whereas negatively correlated with miR-4270 which was consistent with the expression of hsa_circ_0032746. Meanwhile, the knockdown of hsa_circ_0032746 also reduced the expression of MCM3 validated by qPCR validation which was consistent with the inhibitory effect of miR‐4270 mimics and the level of protein expression was reduced on western blot analysis. The co-transfection of sh-circ0032746 and miR-4270 inhibitor increased the protein level of MCM3 compared to sh-circ0032746.

## Conclusion

Hence, we found that hsa_circ_0032746 was upregulated in ESCC and acted as a competing endogenous RNA of miR-4270. Our research suggests that hsa_circ_0032746 functions as a ceRNA to regulate MCM3 expression by sponging miR‐4270. Circ_0032746/miR-4270/MCM3 axis may play an important role in the pathogenesis of ESCC indicating that the further study on circ_0032746 would put more light on clinical significance for the diagnosis and treatment of ESCC and other diseases in the future.
